# Indicator Layers Based on Ethylene-Vinyl Acetate Copolymer (EVA) and Dicyanovinyl Azobenzene Dyes for Fast and Selective Evaluation of Vaporous Biogenic Amines

**DOI:** 10.3390/s18124361

**Published:** 2018-12-10

**Authors:** Tinkara Mastnak, Aleksandra Lobnik, Gerhard J. Mohr, Matjaž Finšgar

**Affiliations:** 1 Institute for Environmental Protection and Sensors, Beloruska 7, SI-2000 Maribor, Slovenia; tinkara.mastnak@ios.si (T.M.); aleksandra.lobnik@ios.si (A.L.); 2 Faculty of Mechanical Engineering, University of Maribor, Smetanova ulica 17, 2000 Maribor, Slovenia; 3 Joanneum Research Forschungsgesellschaft mbH—Materials, Franz-Pichler-Straße 30, A-8160 Weiz, Austria; gerhard.mohr@joanneum.at; 4 Faculty of Chemistry and Chemical Engineering, University of Maribor, Smetanova ulica 17, 2000 Maribor, Slovenia

**Keywords:** azobenzene dye, ethylene-vinyl acetate copolymer, absorption, colorimetry, biogenic amines

## Abstract

The article presents naked-eye methods for fast, sensitive, and selective detection of isopentylamine and cadaverine vapours based on 4-*N*,*N*-dioctylamino-4′-dicyanovinylazobenzene (CR-528) and 4-*N*,*N*-dioctylamino-2′-nitro-4′-dicyanovinylazobenzene (CR-555) dyes immobilized in ethylene-vinyl acetate copolymer (EVA). The reaction of CR-528/EVA and CR-555/EVA indicator layers with isopentylamine vapours caused a vivid colour change from pink/purple to yellow/orange-yellow. Additionally, CR-555/EVA showed colour changes upon exposure to cadaverine. The colour changes were analysed by ultraviolet–visible (UV/VIS) molecular absorption spectroscopy for amine quantification, and the method was partially validated for the detection limit, sensitivity, and linear concentration range. The lowest detection limits were reached with CR-555/EVA indicator layers (0.41 ppm for isopentylamine and 1.80 ppm for cadaverine). The indicator layers based on EVA and dicyanovinyl azobenzene dyes complement the existing library of colorimetric probes for the detection of biogenic amines and show great potential for food quality control.

## 1. Introduction

Sensors are devices that obtain a physical quantity and convert it into an analytical signal (i.e., mechanical, optical, or electrical). The field of sensor technology is extremely broad and its current development is primarily based on simplicity, rapid response, and cost-effectiveness. Food safety is a scientific area that requires advanced handling, storage, and preparation. The primary techniques for food analysis are time-consuming and laborious, whereas sensors and probes offer a faster, easier, functional, and more economical solution.

Azobenzene, with two phenyl rings separated by an azo (–N=N–) bond, serves as the parent molecule for a broad class of aromatic azo compounds. The strong electronic absorption maximum can be tailored by ring substitution, allowing the chemical fine-tuning of colour [1]. Azobenzenes have found applications in the field of nonlinear optics [2], surface relief gratings [3], photo switching [4], optical data storage [5], photoprobes [6], and liquid crystal alignment [7]. They have been extensively investigated as small molecules, as pendants on other molecular structures, or embedded in a solid matrix. Doping azobenzenes into polymer matrices is a convenient inclusion technique that ensures the stability, rigidity, and processability of the system [8]. The non-toxicity of sensor chemistry is an important issue, and while azo dyes are still widely used in textiles, they are frequently banned from use in the food industry because they have been identified as possible allergens [9] and their negative effects on children diagnosed with attention deficit hyperactivity disorder (ADHD) have been reported [10].

Ethylene-vinyl acetate copolymer (EVA) is a non-toxic, insoluble, biocompatible thermoplastic copolymer of ethylene and vinyl acetate [11], and is approved by the FDA for indirect contact [12]. Polar vinyl acetate units in EVA are randomly dispersed in the backbone, which provides excellent flexibility, fracture toughness, light transmission properties, and adhesion to other materials [13]. EVA is used in a variety of commercial applications, such as stent coatings (Bravo Matrix^®^) [14], intravitreal implants (Iluvien^®^, Vitrasert^®^) [15], contraceptive implants (Nexplanon^®^/Implanon NXT^®^) [16], contraceptive intravaginal rings (NuvaRing^®^) [17], and periodontal fibres (Actisite^®^) [18].

Biogenic amines are nitrogenous organic compounds that may occur naturally in foods and beverages, particularly in products that involve a ripening or fermentation period, such as meat products, fish, cheese, beer, and wine [19]. They are generated either as a result of endogenous amino acid decarboxylase activity in raw food material or by the growth of decarboxylase-positive microorganisms under conditions favourable to enzyme activity. Although they are involved in important metabolic and physiological functions in every living organism, consumption at high concentrations may cause undesirable physiological effects [20]. The control of biogenic amines in foods is gaining importance in order to monitor production processes, to ascertain their quality and freshness, and to monitor food safety [21].

A variety of amine sensors and probes have been developed based on different sensing mechanisms. Among these, optical approaches can provide rapid, practical, and sensitive identification of an extensive range of organic vapours, which is why they have practical significance in a broad range of fields [22]. Colorimetric sensing systems are particularly attractive because they are relatively simple, inexpensive, and do not require any power supply. Moreover, the response signals can be detected with the naked eye or recorded with a conventional scanner or camera. Another important advantage of colorimetric sensors and probes is the possibility to base them on an array of chromogenic reagents for simultaneous detection of many closely related analytes [23].

Some examples of colorimetric sensors and probes for the detection of different biogenic amines are presented in Table 1. One of the major approaches to the colorimetric detection of volatile amines consists of pH indicators embedded in a solid support or a matrix. For example, Sun et al. improved the sensitivity of pH indicators by making ambient pH values reach their colour transition points in the sol-gel system and fabricated a sensor array for the determination of ethylenediamine [24]. On the other hand, Bueno et al. immobilized five different pH indicators on cellulose acetate membranes and constructed a plastic device for discriminating between different types of amines (triethylamine, isobutylamine, isopentylamine) [25]. Furthermore, Schaude et al. developed a sensor layer by covalently immobilizing a pH indicator dye on cellulose microparticles and embedding them in food-grade silicone. The system was used to monitor the volatile basic nitrogen during meat spoilage [26].

Instead of pH indicators, porphine dyes can also be used, as shown by Tang et al. In their preliminary study, six chemically responsive porphine dyes were impregnated on an inert substrate plate and used for the qualitative discrimination and quantitative detection of volatile amines. The best results were obtained for trimethylamine, reaching an LOD lower than 50 ppb [27]. Another type of dye was used by Sutarlie and Yang. They doped several transparent polymers with iron phthalocyanine (FePc) and studied their colorimetric responses to hexylamine. Although they all exhibited colour changes upon exposure to this analyte, only poly(dimethylsiloxane) doped with FePc showed a selective and reversible response [23]. Alternatively, Xiao-Wei et al. created disposable colorimetric arrays of natural pigments by printing them on reverse phase silica gel plates. A biogenic amine index (BAI) for the determination of meat freshness was developed from the sum of putrescine and cadaverine and successfully applied in the determination of pork spoilage [28]. An interesting approach was taken by Dudnyk et al. [31]. They developed an edible sensor film based on pectin and red-cabbage extract. The sensor was exposed to a series of synthetic amines, including ammonia, cadaverine, and pyridine, and showed colour changes from purple to yellow. When exposed to the headspace above beef, chicken, shrimp, or whiting (fish), the sensors showed visual changes as the food samples degraded. In another study, 16 chemo-sensitive compounds were applied on silica gel plates and used for colorimetric detection of trimethylamine, dimethylamine, cadaverine, and putrescine. The colorimetric sensor array was then used to monitor fish spoilage over time [29]. Lin et al. prepared thin films from solutions of 4-(dioctylamino)-4′-(trifluoroacetyl)azobenzene in tetrahydrofuran deposited on polyethylene terephthalate (PET) foil. A distinct optical response of the sensors exposed to ammonia, tetramethylammonium hydroxide, ethylamine, cadaverine, and putrescine was observed [30]. A completely different approach was presented in a study where visual monitoring of biogenic amines was achieved with a portable sensory hydrogel made from Au nanorods and agarose. The multiple colour changes of the sensory hydrogel were found to correlate with the biogenic amines generated during the storage of foodstuffs, thus enabling in situ monitoring of food spoilage [32].

We have previously reported on the synthesis of two new azobenzene dyes, 4-*N*,*N*-dioctylamino-4′-dicyanovinylazobenzene (CR-528) and 4-*N*,*N*-dioctylamino-2′-nitro-4′-dicyanovinylazobenzene (CR-555), and their detection of biogenic amines in ethanol solution, which exhibited significant colour changes from pink/purple to pale yellow/orange-yellow [33]. Based on our previous work, we prepared two different types of indicator layers by immobilizing CR-528 and CR-555 on EVA and studied their response to the vapours of several biogenic amines. CR-528/EVA and CR-555/EVA both showed excellent sensitivity to isopentylamine, but only CR-555/EVA showed colour changes upon the addition of cadaverine. Hitherto, to the best of our knowledge, these are the first examples of colorimetric probes for the detection of biogenic amines in the vapour phase based on EVA and azobenzene dyes.

## 2. Materials and Methods

### 2.1. Chemicals and Starting Materials

Diethylamine, ethanolamine, isopentylamine, spermidine, triethylamine, tetrahydrofuran (THF), and EVA (vinyl acetate 40 wt. %) chemicals of analytical grade were supplied by Sigma Aldrich (St. Louis, MO, USA).

### 2.2. Materials Characterization

Fourier-transform infrared (FTIR) spectra were recorded on a Spectrum Two FT-IR Spectrometer (Perkin-Elmer, Waltham, MA, USA). The morphology of the probes was observed by two different scanning electron microscopes (SEM, FEI Sirion 400 NC and FEI Quanta 200 3D) at an acceleration voltage of 15.0 kV. The ultraviolet–visible (UV-VIS) molecular absorption spectra were recorded with a Lambda 35 UV-Vis spectrometer (Perkin-Elmer, Waltham, MA, USA) at 20 ± 1 °C using polystyrene cuvettes with 1 cm path length.

### 2.3. Fabrication of Indicator Layers

4-*N*,*N*-dioctylamino-4′-dicyanovinylazobenzene (CR-528) and 4-*N*,*N*-dioctylamino-2′-nitro-4′-dicyanovinylazobenzene (CR-555) were synthetized as described previously [33]. The EVA solution was prepared by dissolving 0.5 g of EVA in 5.0 mL of THF and stirred overnight. 1.5 mL of the solution was added to a glass vial containing 2.0 ± 0.1 mg of azo dye and stirred until the dye completely dissolved (approximately 30 min). The mixture was then knife-coated onto transparent polyethylene terephthalate foil (PET, Mylar) by using a double bar film applicator (BYK) and left to dry at ambient conditions for 1 h. The indicator layers thus prepared were kept in sealed plastic containers, away from direct sunlight and the laboratory environment, and were stable for at least 6 months.

### 2.4. Experimental Procedure

Indicator layers were cut into small pieces suitable for polystyrene cuvettes with a 1 cm path, and attached to a glass slide that could fit into a 20.0 mL headspace vial (Supelco, Bellefonte, PA, USA). After tightly closing the vial with a magnetic screw cap, the required volume of the vaporous analyte was injected with a Gastight syringe (Hamilton, Reno, NV, USA). After 1 min, the cap was removed and the vial was left under a fume hood to enable complete evaporation of the analyte. The indicator layer was then carefully removed from the glass slide, attached to a polystyrene cuvette, covered with a plastic cap, and the absorbance was measured. The described procedure was possible because the analyte recognition in the process is irreversible. A new blank was recorded for each set of measurements. All measurements were performed in triplicate.

## 3. Results and Discussion

### 3.1. Preparation of Indicator Layers

For the purpose of indicator layer preparation, an appropriate transparent solid-state platform (substrate) was chosen for the deposition of the EVA and azobenzene dye (CR-528 or CR-555) mixture. A substrate is often a flat surface on which sensing material is processed to create a sensor or a probe [34]. For the fabrication of probes intended for long-term use, the substrates need to be resilient and have a long lifetime. Additionally, low cost and simple processing are often critical factors. These requirements are met by polymeric substrates, especially polyester films known as PET [35]. Mylar™ is a special type of biaxially-oriented PET, which was developed to achieve better mechanical durability, tensile strength, and chemical and dimensional stability, thus enhancing the sensing performance [36]. For the controlled deposition of our liquid material, knife coating was selected due to its simplicity, great material efficiency, low cost production capability, and reproducibility [37]. It has been successfully applied in a roll-to-roll process for the manufacture of polymer solar cells [38].

### 3.2. Spectroscopic Measurements

**Figure 1:**
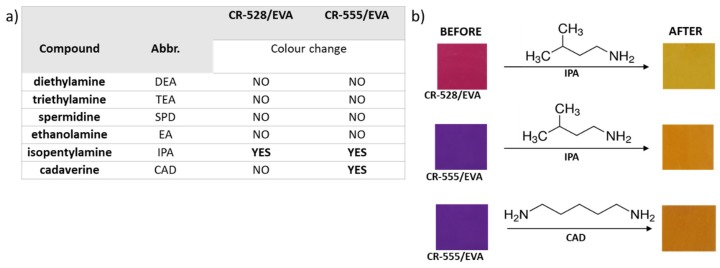
(**a**) A table presenting the list of compounds used in the preliminary experiment for qualitative discrimination between vaporous biogenic amines. (**b**) The observed colour change before and after the exposure of the CR-528 indicator layer to isopentylamine and the CR-555 indicator layer to isopentylamine and cadaverine.

In a preliminary experiment, the fabricated indicator layers, CR-528/EVA and CR-555/EVA, were explored as colorimetric devices for determining the presence of biogenic amines in the vapour phase. A series of amines were chosen, as listed in Figure 1a, and their saturated vapours were prepared by boiling 200 µL of each compound in a tightly sealed headspace vial. For qualitative discrimination, the layers were exposed to the saturated vapours by simply laying them over the vial containing the analyte immediately after opening. The results showed that the reaction of CR-528/EVA and CR-555/EVA with isopentylamine vapours caused a distinct colour change from pink/purple to yellow/orange-yellow (see Figure 1), but only CR-555/EVA showed a colour change upon exposure to cadaverine.

To determine the potential cross sensitivity of CR-528/EVA and CR-555/EVA indicator layers to vapours of different organic solvents, the probes were exposed to saturated vapours of acetone, hexane, ethyl acetate, ethanol, and acetonitrile. There were no major changes in the absorbance values before and after the exposure of CR-528/EVA and CR-555/EVA probes to the saturated vapours of aforementioned organic solvents (results are presented in Figures S1 and S2 in the Supporting information).

The synthetic and optical properties of CR-528 and CR-555 have already been described in detail [33]. In short, the probes differ in colour and, consequently, the absorption maxima due to the difference in the chemical structures of the indicator dyes, and the blue shift is caused by the irreversible addition reaction between the nucleophilic amine and dicyanovinyl groups. The absorption maxima of the azo dyes upon their incorporation into the EVA slightly changed compared to their absorption maxima in ethanol solution. In the case of CR-528, the peak shifted from 528 nm to 530 nm, whereas in the case of CR-555, the peak shifted from 555 nm to 570 nm. This effect is probably caused by the markedly different solvatochromism of the dyes in EVA.

**Figure 2:**
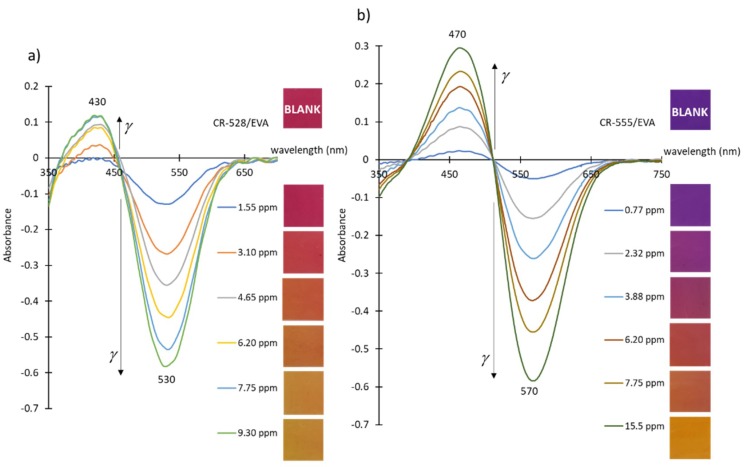
(**a**) Spectral changes with corresponding colour changes after the exposure of the CR-528/EVA layer to increasing concentrations of isopentylamine vapours. (**b**) Spectral changes with corresponding colour changes after the exposure of the CR-555/EVA layer to increasing concentrations of isopentylamine vapours.

In order to quantitatively evaluate the changes in the absorbance of the CR-528/EVA and CR-555/EVA layers, we conducted a series of experiments where a new piece of probe was used for each measurement. The spectra acquired from the absorbance measurements of the CR-528/EVA layer after the addition of increasing concentrations of isopentylamine vapours show a large decrease in the absorbance intensity at 530 nm and a much less significant increase at 430 nm. The negative absorbance values of the peaks at 530 nm (see Figure 2) stem from the fact that our blank was the original indicator layer and the decrease in the absorbance corresponds to the decreased intensity of the original (pink) colour of CR-528 in EVA. At the same time, the signal at 430 nm increased due to the increase in yellow colouring. The spectra corresponding to the absorbance of CR-555/EVA after the exposure of increasing concentrations of isopentylamine vapours show similar characteristics. The peaks at 570 nm have negative values (see Figure 2) due to the decreased intensity of the original (purple) colour of CR-555 in EVA. The simultaneous increase in the absorbance intensity at 470 nm can be attributed to the increase in orange-yellow colouring. Generally, signal changes at shorter wavelengths (below the isosbestic point) are smaller than at longer wavelengths (above the isosbestic point), because the molar extinction coefficients of the dyes are higher for the dicyanovinyl derivative than for the reaction product. The colorimetric responses and their statistical evaluation are presented in detail in Section 3.3.

### 3.3. Analytical Method Validation

For the determination of the isopentylamine and cadaverine concentration, UV/VIS molecular absorption spectroscopy and calibration curves were employed [39]. Partial method validation was performed by determining the LOD, linear concentration range, confidence intervals, and sensitivity. Possible outliers for the replicate absorbance measurements using new sensors at single concentration points were checked by means of Grubbs’ and Dixon’s outlier tests [40]. If an outlier was present, this particular value was discarded and not used for the calculation of the average absorbance.

**Figure 3:**
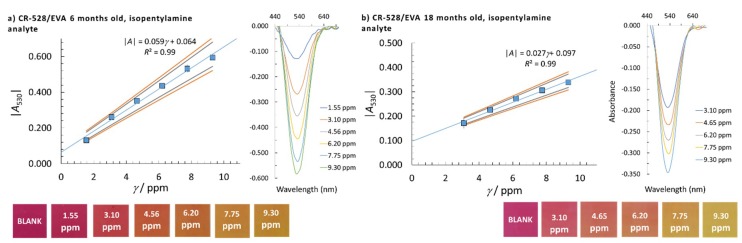
Linear concentration range with corresponding colour changes, confidence intervals (90% and 95% confidence), and molecular absorption spectra of isopentylamine for the CR-528/EVA indicator layer; (**a**) six months and (**b**) 18 months old. The analytical signal was measured at λ = 530 nm.

**Figure 4:**
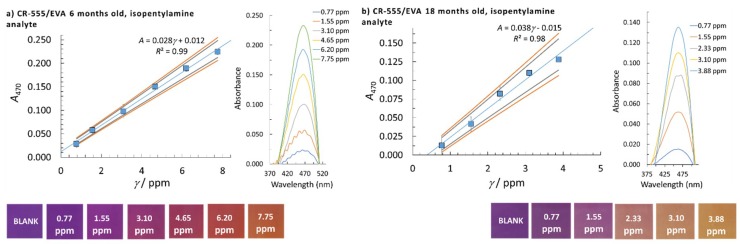
Linear concentration range with corresponding colour changes, confidence intervals (90% and 95% confidence), and molecular absorption spectra of isopentylamine for the CR-555/EVA indicator layer; (**a**) six months and (**b**) 18 months old. The analytical signal was measured at λ = 470 nm.

**Figure 5:**
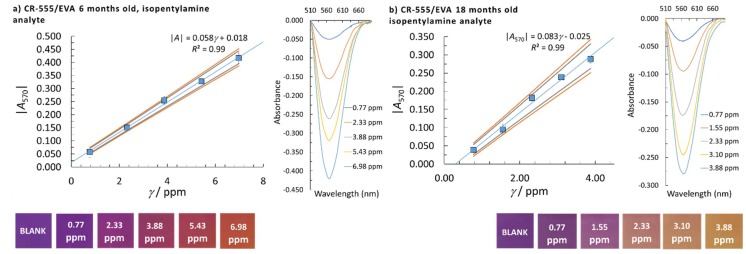
Linear concentration range with corresponding colour changes, confidence intervals (90% and 95% confidence), and molecular absorption spectra of isopentylamine for the CR-555/EVA indicator layer; (**a**) six months and (**b**) 18 months old. The analytical signal was measured at λ = 570 nm.

**Figure 6:**
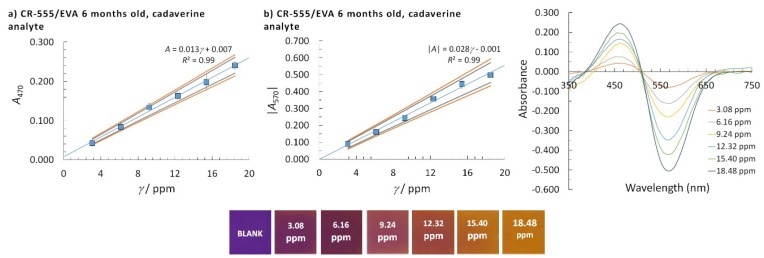
Linear concentration range with corresponding colour changes, confidence intervals (90% and 95% confidence), and molecular absorption spectra of cadaverine for the CR-555/EVA indicator layer. The analytical signal was measured at (**a**) λ = 470 nm and (**b**) λ = 570 nm.

The LOD was determined as LOD = 3·*s*/*b*_1_ (*s* is the standard deviation and *b*_1_ is the calibration curve slope), where *s* was determined as *s*_e_ (residual standard deviation) or *s*_b0_ (standard deviation of the intercept) according to Equations (1) and (2):

(1) $$s_{e}=\sqrt{\frac{\sum_{j=1}^{k}\left(y_{j}-{\hat{y}}_{j}\right)2}{k-2}}$$

(2) $$s_{b0}=\;s_{e}\sqrt{\frac{\sum_{j=1}^{k}x_{j}^{2}}{k\left(\sum_{j=1}^{k}x_{j}^{2}-\frac{1}{k}{\left(\sum_{j=1}^{k}x_{j}\right)}^{2}\right)}}$$

where $y_{j}$ is the average absorbance for a certain calibration point, ${\hat{y}}_{j}$ is the model absorbance, k is the number of calibration points, and $x_{j}$ is the concentration of a certain calibration point. The highest calculated LOD by using *s*_e_ or *s*_b0_ was taken as the method’s LOD (the worst-case scenario of the method used). To determine the linear concentration range, at least five calibration points were employed and at least three replicate measurements were performed at a single calibration point. The homogeneity of the variances at different calibration points was checked using Bartlett’s and Cochran’s tests, which was needed to accept the calibration points for the reported linear concentration range. The normality of the data distribution was confirmed by the quantile-quantile plot. The square of the correlation coefficient, *R*^2^, needed to be higher than 0.99 to accept the linearity and the quality coefficient (*QC*) needed to be equal to or lower than 5.0%. The sensitivity was evaluated from the calibration curve slopes. A summary of the analytical performances of the CR-528/EVA and CR-555/EVA indicator layers that were six or 18 months old for isopentylamine and cadaverine determination are given in Table 2 and Figure 3, Figure 4, Figure 5 and Figure 6.

The absorbance analysis at λ = 530 nm with CR-528/EVA shows a linear response for isopentylamine in the 1.55 ppm–9.30 ppm mass concentration range for the six-month-old probe. This linear response becomes narrower when performing measurements with CR-528/EVA aged for 18 months (3.10 ppm–9.30 ppm). For the latter, the LOD increases and the sensitivity (calibration curve slope) decreases. It has to be noted that an absolute value of the absorbance was taken as an analytical signal, since the absorbance values at this λ are negative due to the reasons explained in Section 3.2.

When CR-555/EVA was used for isopentylamine determination and analysis performed at λ = 470 nm, lower LODs and lower linear concentration limits were obtained in comparison with the results for CR-528/EVA (Table 2). Moreover, the statistical analysis of the data at λ = 470 nm for the 18-month-old CR-555/EVA indicator layer causes a decrease in the LOD and a drop in the *R*^2^ value to 0.98 (see Figure 4). Furthermore, when the analysis with CR-555/EVA was performed at λ = 570 nm, the lowest LODs for isopentylamine determination were obtained among all sets of data, i.e., 0.44 ppm and 0.41 ppm when using six- and 18-month-old CR-555/EVA probes, respectively (Figure 5).

Cadaverine analysis was possible at two wavelengths, namely 470 nm and 570 nm, but only with CR-555/EVA (Figure 6). For both sets of data, the LOD values and linear concentration ranges were similar.

The above described results are in accordance with the data presented in our previous paper [33]. In brief, the nitro acceptor group in the CR-555 reactive dye contributes to the higher sensitivity and lower detected analyte concentrations. This structural difference in dicyanovinyl azobenzene dyes is therefore the reason for the difference in the LODs for isopentylamine, where the best value obtained for CR-555/EVA is more than two-times lower than the best value obtained for the CR-528/EVA indicator layer. The lack of a nitro acceptor group can also explain the inability of the CR-528 dicyanovinyl dye to react with cadaverine under these conditions. The sensing performances of 18-month-old probes proved that our system has outstanding shelf-life stability, which is very important when considering applications in smart food packaging.

### 3.4. Characterisation of Indicator Layers

The morphology of the functional layers is important for their physical properties and sensing performance. Furthermore, the potential structural changes caused by ageing might have a significant effect on the stability of the indicator layers. The stability of the probe depends on the indicator dye and the matrix. The latter protects the indicator layer from degradation by acting as a chemical barrier and protects the dye from potential photodegratory reactions [41]. To shed light on the reaction mechanism and surface properties of the CR-528/EVA and CR-555/EVA layers, we characterized them by means of FTIR spectroscopy and scanning electron microscopy, as presented in Section 3.4.1 and Section 3.4.2, respectively.

#### 3.4.1. FTIR Analysis

**Figure 7:**
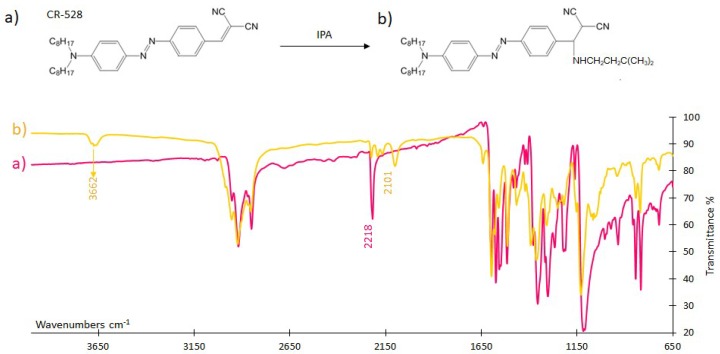
CR-528 CR-528 (**a**) before and (**b**) after exposure to saturated vapours of isopentylamine. The formation of an amino signal at 3661 cm^−1^, the decrease in the nitrile vibration at 2227 cm^−1^, and the signal at 2103 cm^−1^ are shown. For a better understanding, the proposed reaction mechanism is depicted.

**Figure 8:**
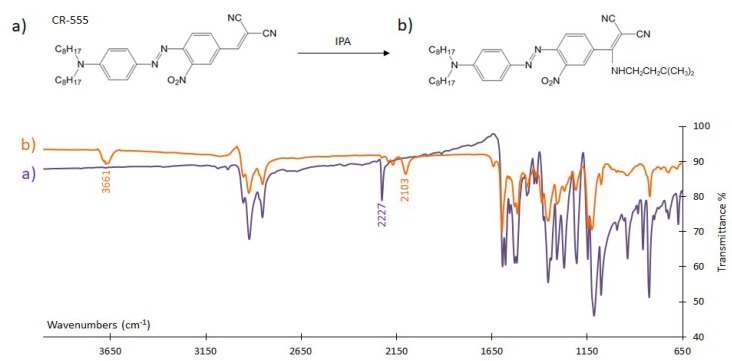
CR-555 (**a**) before and (**b**) after the exposure to saturated vapors of isopentylamine. The formation of an amino signal at 3662 cm^−1^, the decrease in the nitrile vibration at 2218 cm^−1^, and the signal at 2101 cm^−1^ are shown. For a better understanding, the proposed reaction mechanism is depicted.

FTIR spectroscopy was used to investigate the interaction mechanism of CR-528 and CR-555 dyes with saturated isopentylamine vapours. Figure 7 and Figure 8 show that the spectra of CR-528 and CR-555 are characterized by several typical signals. Signals in the range from 2930 cm^−1^–2850 cm^−1^ are attributed to aliphatic C-H stretching, whereas the signals at 2218 cm^−1^ (for CR-528) and at 2227 cm^−1^ (for CR-555) represent nitrile stretching. The interaction of vaporous isopentylamine with the azo dyes causes the formation of an amino signal at a wavenumber of around 3660 cm^−1^, which was attributed to the formation of a secondary amine. Simultaneously, the nitrile signals at 2218 cm^−1^ for CR-528 and 2227 cm^−1^ for CR-555 decrease significantly and a new signal for the nitrile group is formed at approximately 2100 cm^−1^. The obtained FTIR spectra confirmed the proposed reaction mechanisms illustrated in Figure 7 and Figure 8, and can also be applied for the interpretation of the reaction mechanism between CR-555 and cadaverine because the second analyte contains similar primary aliphatic amino groups to isopentylamine.

#### 3.4.2. Morphology of Indicator Layers

**Figure 9:**
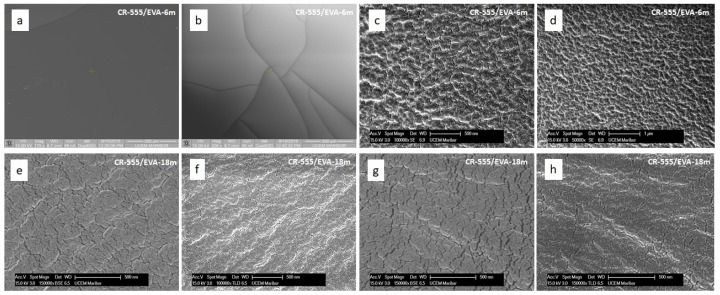
SEM images of CR-555/EVA recorded with FEI Quanta 200 3D (**a**,**b**) and FEI Sirion 400 NC (**c**–**h**) showing the surface of the indicator layer. (**a**–**d**) The surface of the CR-555/EVA layers aged for six months. (**e**–**h**) The CR-555/EVA layers aged for 18 months. Microphotographs were taken at different magnifications and with different detection methods.

To gain a better understanding of the morphology and structure of the sensing probes, we obtained several SEM top view images of the CR-555/EVA indicator layer at different magnifications (Figure 9). The SEM analysis revealed that the surface of CR-555/EVA was homogeneous although rough and without any degradation, thus confirming the adequacy of the knife-coating technique for the reproducible fabrication of uniform layers. Moreover, the SEM images of the CR-555/EVA layer aged for 18 months exhibited the exact same morphology as its 12-month-younger equivalent, thus suggesting the excellent shelf-life stability of the EVA-based indicator layers.

It has to be noted that the original indicator layer was cut into dozens of pieces that were subsequently used for the absorbance measurements, so a single SEM image reflects the characteristics of a larger number of sensing probes. Furthermore, there is no difference in the morphology and structure of the CR-528/EVA and CR-555/EVA indicator layers.

## 4. Conclusions

To address the important issue of toxicity, CR-528 and CR-555 azo dyes were evaluated by comparing the possible products of their reductive cleavage with the compounds listed in EU Directive 2002/61/EC [42]. We found that the dyes used in this study meet the requirements. However, to enable their use in food packaging, further studies concerning leaching need to be carried out by testing the indicator layers against common extracting solvents, such as water, ethanol, acetic acid, and olive oil [43].

To conclude, we prepared two different types of indicator layers by immobilizing CR-528 and CR-555 dicyanovinyl dyes on EVA and studied their response to the vapours of several biogenic amines. UV/VIS molecular absorption spectroscopy was employed for the determination of analyte concentrations. Partial method validation was performed by determining the LOD, linear concentration range, confidence intervals, and sensitivity. CR-528/EVA and CR-555/EVA probes showed excellent sensitivity to isopentylamine, but only CR-555/EVA showed characteristic colour changes upon the addition of cadaverine. The spectra obtained with the CR-555/EVA indicator layers were statistically analysed at two different wavelengths, whereas the results obtained with the CR-528/EVA indicator layers provided data for the analysis at a single wavelength. The lowest detection limit (0.41 ppm) was achieved with the CR-555/EVA probe and isopentylamine analyte when the analysis was performed at 570 nm. The shelf life stability of the indicator layers based on EVA and dicyanovinyl azobenzene dyes is exceptional, which makes them promising candidates for applications in smart packaging and food quality control.

## Figures and Tables

**Table 1:** Summary of colorimetric sensors and probes for different biogenic amines indicating their chemical composition, response time, application, and limit of detection (LOD).

Sensor/Probe—Matrix/Support	Analyte	Response Time	Application	LOD	Ref.
pH indicators—sol-gel	ethylenediamine gas	30 min	Fish freshness	10 ppm	[24]
pH indicators—cellulose acetate membranes	triethylamine, isobutylamine, isopentylamine vapours	10 min	Meat spoilage	1–5 ppm	[25]
iron(II) phthalocyanine—poly(dimethylsiloxane)	Saturated * hexylamine vapours	15 min	Environmental monitoring	11,600 ppm *	[23]
chemically sensitive dyes—inert substrate plate	Trimethylamine vapours	15 min	Preliminary study	50 ppb	[27]
pH indicator dye—cellulose microparticles	total volatile basic nitrogen (TVB-N)	1.5 h	Meat spoilage	Not given	[26]
Natural pigments—silica gel plates	cadaverine, putrescine in pork	3 days	Pork spoilage	4 ppm	[28]
16 chemo-sensitive compounds—silica gel plates	trimethylamine, dimethylamine, cadaverine, putrescine	2–6 h	Fish spoilage	20–200 ppm	[29]
4-(dioctylamino)-4′-(trifluoroacetyl)azobenzene in tetrahydrofuran—PET foil	cadaverine	2 h	Food spoilage	Not given	[30]
red-cabbage extract and pectin	cadaverine	1 day	Meat and seafood freshness	1 ppm	[31]
Au nanorods—agarose	trimethylamine	3 h	Food spoilage	Not given	[32]

* The authors in the cited reference conducted experiments with saturated vapours, which is the reason for the drastically higher LOD value.

**Table 2:** LOD and linear concentration ranges for CR-528/EVA and CR-555/EVA probes measured at different λ.

	CR-528/EVA		CR-555/EVA				CR-555/EVA	
Analyte	Isopentylamine		Isopentylamine				Cadaverine	
**Probe age/months**	6	18	6	18	6	18	6	6
**Detection at λ/nm**	530	530	470	470	570	570	470	570
**LOD/ppm**	0.98	1.23	0.61	0.54	0.44	0.41	1.86	1.80
**Linear concentration range/ppm**	1.55–9.30	3.10–9.30	0.77–7.75	0.77–3.88*	0.77–6.98	0.77–3.88	3.08–18.48	3.08–18.48
**Calibration curve slope/ppm^–1^**	0.059	0.027	0.028	0.038	0.058	0.083	0.013	0.028

* *R*^2^ < 0.99.

## Supplementary Materials

The following are available online at http://www.mdpi.com/1424-8220/18/12/4361/s1, Figure S1: Spectral changes after the exposure of (a) CR-528/EVA layers and (b) CR-555/EVA layers to saturated vapours of different organic solvents, i.e., acetone, hexane, ethyl acetate, ethanol, and acetonitrile. Figure S2: Changes in absorbance values after the exposure of (a) CR-528/EVA layers and (b) CR-555/EVA layers to the saturated vapours of different organic solvents, i.e. acetone, hexane, ethyl acetate, ethanol, and acetonitrile. The signal was measured at λ = 530 nm for CR-528/EVA layers and at λ = 470 nm and 570 nm for CR-555/EVA layers.
