# Supplementary material for: Indicator Layers Based on Ethylene-Vinyl Acetate Copolymer (EVA) and Dicyanovinyl Azobenzene Dyes for Fast and Selective Evaluation of Vaporous Biogenic Amines
Source: Sensors (Basel). 2018 Dec 10;18(12):4361. doi: 10.3390/s18124361 (PMC6308792; doi:10.3390/s18124361)
Supplement: Supplementary file 1 [file sensors-18-04361-s001.pdf]

Supplementary information

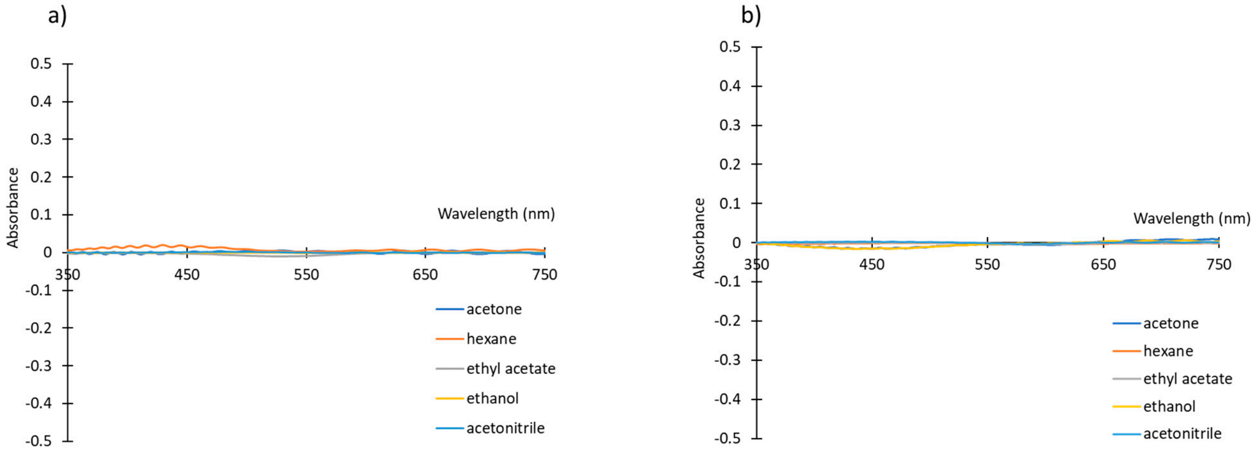

Figure S1: Spectral changes after the exposure of a) CR-528/EVA layers and b) CR-555/EVA layers to saturated vapours of different organic solvents, i.e. acetone, hexane, ethyl acetate, ethanol, and acetonitrile.

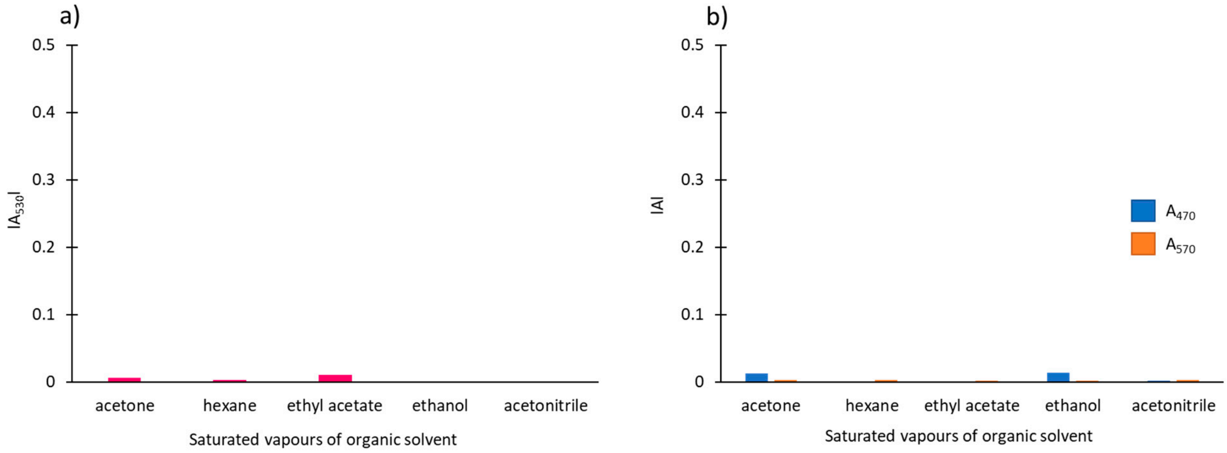

Figure S2: Changes in absorbance values after the exposure of a) CR-528/EVA layers and b) CR-555/EVA layers to the saturated vapours of different organic solvents, i.e. acetone, hexane, ethyl acetate, ethanol, and acetonitrile. The signal was measured at λ= 530 nm for CR-528/EVA layers and at λ = 470 nm and 570 nm for CR-555/EVA layers.
